# The SCottish Alcoholic Liver disease Evaluation: A Population-Level Matched Cohort Study of Hospital-Based Costs, 1991-2011

**DOI:** 10.1371/journal.pone.0162980

**Published:** 2016-10-26

**Authors:** Janet Bouttell, James Lewsey, Claudia Geue, Grace Antony, Andrew Briggs, Gerry McCartney, Sharon Hutchinson, Lesley Graham, Mathis Heydtmann

**Affiliations:** 1 Health Economics and Health Technology Assessment, University of Glasgow, Glasgow, United Kingdom; 2 NHS Health Scotland, Glasgow, United Kingdom; 3 School of Health and Life Sciences, Glasgow Caledonian University, Glasgow, United Kingdom; 4 Information Services Division, NHS National Services Scotland, Edinburgh, United Kingdom; 5 Department of Gastroenterology, Royal Alexandra Hospital, Paisley, United Kingdom; The Chinese University of Hong Kong, HONG KONG

## Abstract

Studies assessing the costs of alcoholic liver disease are lacking. We aimed to calculate the costs of hospitalisations before and after diagnosis compared to population controls matched by age, sex and socio-economic deprivation. We aimed to use population level data to identify a cohort of individuals hospitalised for the first time with alcoholic liver disease in Scotland between 1991 and 2011.Incident cases were classified by disease severity, sex, age group, socio-economic deprivation and year of index admission. 5 matched controls for every incident case were identified from the Scottish population level primary care database. Hospital costs were calculated for both cases and controls using length of stay from morbidity records and hospital-specific daily rates by specialty. Remaining lifetime costs were estimated using parametric survival models and predicted annual costs. 35,208 incident alcoholic liver disease hospitalisations were identified. Mean annual hospital costs for cases were 2.3 times that of controls pre diagnosis (£804 higher) and 10.2 times (£12,774 higher) post diagnosis. Mean incident admission cost was £6,663. Remaining lifetime cost for a male, 50–59 years old, living in the most deprived area diagnosed with acoholic liver disease was estimated to be £65,999 higher than the matched controls (£12,474 for 7.43 years remaining life compared to £1,224 for 21.8 years). In Scotland, alcoholic liver disease diagnosis is associated with significant increases in admissions to hospital both before and after diagnosis. Our results provide robust population level estimates of costs of alcoholic liver disease for the purposes of health-care delivery, planning and future cost-effectiveness analyses.

## Introduction

Alcoholic Liver Disease (ALD) accounts for significant morbidity, mortality and economic burden worldwide[1]. The UK has one of the highest rates of ALD in Western Europe[2,3] and Scotland is associated with substantially higher rates of alcohol consumption (as measured by alcohol sales) and alcohol-related harms than the rest of the UK[4]. There is much uncertainty surrounding trends and the level of burden associated with ALD across Europe [5,6] as comparisons are hindered by the complex aetiology of liver disease [5], issues with late diagnosis and under-reporting of alcohol as a contributing factor [5]. Studies looking at the costs associated with ALD have estimated either population level costs [7,8] or episode based costs [9,10] and have not distinguished incident cases from readmissions. To our knowledge there are no studies which examine the costs associated with individual cases of ALD nor any costs studies using matched controls.

In this study, costs and survival data were modelled to allow annual costs per incident case and remaining lifetime costs to be estimated. In addition we estimated costs of incident admission according to disease severity. Use of population level data and the comparison with matched controls enabled us to produce a robust estimate of the additional hospital costs of an incident ALD case in Scotland. High quality cost of illness studies are not only valuable for health-care policy makers and planners, but also provide valuable information for future cost-effectiveness studies of interventions that aim to prevent incident cases or improve individual prognosis.

This SCottish Alcoholic Liver disease Evaluation (SCALE) study aimed to identify hospital admissions and associated costs before and after diagnosis and outcomes for all individuals hospitalised for the first time with ALD in Scotland between 1991–2011 compared to population controls matched by age, sex and socio-economic deprivation.

## Materials and Methods

### Data sources

#### Hospital admissions data

Admissions data were drawn from the Scottish Morbidity Records (SMR01 and SMR04), which are national data schemes that record comprehensive information relating to all inpatient and day cases admitted to acute and psychiatric National Health Service (NHS) hospitals in Scotland [11]. Several hospital episodes may be included within one continuous in-patient stay (CIS). We obtained data for both episodes and CIS as the episodes detail the hospital and type of care provided and CIS allows consecutive or concurrent episodes to be linked into one continuous admission (stay).

#### Study population

We obtained anonymised individual level data on all hospital admissions for patients who were admitted with ALD in a principal or secondary diagnostic position on at least one occasion between 1981 and 2011, including sex, age group and Scottish Index of Multiple Deprivation (SIMD) quintiles from Information Services Division (ISD), NHS National Services Scotland (NSS). SIMD measures deprivation using a combination of income, employment, health, education, skills and training, housing, geographic access and crime indicators. ALD admissions were classified using the International Classification of Diseases (ICD9/ICD-10) codes set used by ISD who are responsible for publishing national statistics on alcohol-related hospital admissions in Scotland (see Table 1). Definitions of severity groupings are shown in Table 2

**Table 1:** Definition of incident ALD hospitalisation disease severity groups (Group: 1) ALD with decompensation (more severe ALD), 2) ALD without decompensation (less severe ALD) and 3) hospitalisation with ALD (but not for ALD)(see Table 2 for definitions of severity groupings).

	Group 1	Group 1	Group 2	Group 3
ALD in principal diagnostic position	x		x	
ALD in secondary diagnostic position		x		x
Decompensation in principal diagnostic position		x		
Decompensation in secondary diagnostic position	x			

**Table 2:** Definitions of severity groupings.

ALD diagnosis codes	ICD 10th Revision ALD codes (9th revision in brackets)—K70.0 (571.0) Alcoholic Fatty Liver, K70.1 (571.1) Alcoholic hepatitis,
	K70.2 Alcoholic fibrosis and sclerosis of the liver, K70.3 (571.2) Alcoholic cirrhosis of the liver, K70.4 Alcoholic hepatic failure,
	K70.9 (571.3) Alcoholic liver disease (unspecified)
Decompensation codes	ICD 10th Revision decomposition codes (9th revision equivalent in brackets)—C22.0 (I55.0) Primary Liver Cancer, I85.0 (456.0)
	Oesophageal Varices (with bleeding), K72.0 (570) Acute and sub-acute failure of the liver, K72.1(572.2) Chronic hepatic
	failure,K72.9 (572.8) Hepatic coma, K76.7 (572.4) Hepatorenal Syndrome, R18 (789.5) Ascites, R17(782.4) Jaundice (>90%
	sensitivity on review of patient records), R402 (780.01) Coma (>75% sensitivity on review of patient records), K766 (5723)
	Portal Hypertension.

#### Incident cases

An incident ALD hospitalisation was defined as ALD in any diagnostic position and no ALD diagnosis was found in hospitalisations for that patient in the preceding 10 years. This length of ‘look back’ period kept the double-counting of cases low (0.7%) while still retaining over 20 years of study period (1991–2011).

#### Controls

The control group was identified from the Community Health Index (a register of all individuals registered with primary care practices in Scotland) [12,13] and comprised 5 individuals per ALD case. Controls were matched to ALD cases with the same age, sex and SIMD area deprivation quintile. The only qualifying conditions for controls was that they were alive at the date of their matched case’s incident hospital admission and had no ALD hospitalisations during 1991–2011. The selection of population controls (rather than those in hospital on the incident date) was motivated by the aim of the study to identify the additional hospital cost of ALD over and above a typical population cohort.

#### Death registry data

Date of death, if applicable, was obtained for all cases and controls who died during the period under review from NRS.

#### Cost data

Dates of admission and discharge from the Scottish Morbidity Records were used to calculate length of stay. This was multiplied by a daily (per diem) cost based on specialty and hospital codes taken from the Scottish Costs Book 2013 [14]. These costs include direct costs, indirect costs and allocated overhead and are calculated as set out in the Scottish Costs Book Health Services Manual [15]. In a 2012 assessment dates of admission and discharge and allocated specialty were found to be over 98% accurate [16].

#### Disease severity

Incident ALD hospitalisations were classified into 3 severity groups as set out in Table 1 following a detailed clinical review of hospital records. We analysed the sensitivity and specificity of including patients presenting with potential decompensation codes (not specific to liver disease: coma, jaundice and hematemesis) in group 1. This allowed an incident ALD hospitalisation to be identified with ALD coded at a secondary diagnostic position.

### Methods

#### Descriptive analysis

We categorised incident admissions by sex, age group, socio-economic deprivation category and co-morbidities for each severity grouping. For analysis of hospital admissions the incident admission date for a case was used to attach a pseudo-date of first ALD event for each of the 5 matched controls. In each 180 day interval for a maximum of 14 years before and after the incident date the percentage of cases and controls hospitalised at least once in that period and the mean cost per person hospitalised were calculated.

#### Statistical analysis

Incident hospitalisation rates for 1991–2011 by sex, age group, year of admission and socio-economic deprivation were modelled using negative binomial regression. Mortality during the incident ALD hospitalisation (which we have termed in-hospital mortality) was modelled using logistic regression to determine adjusted effects on mortality of the same covariates as used for incident rates as well as the patient’s co-morbidities. For patients discharged alive, time to all-cause death after incident hospitalisation was modelled using Cox regression again with the same set of covariates including co-morbidities and with year of discharge replacing year of admission. The number of readmissions per year was calculated, accounting for different exposure lengths caused by death and the end of follow-up period, and these rates were modelled using negative binomial regression with the same set of covariates as for modelling in-hospital mortality. Costs were analysed using generalised linear models (GLMs) with gamma distribution and a log link with the covariates time period, sex, age group, socio-economic deprivation category and year of admission. Despite matching on sex, age group and socio-economic deprivation category we adjusted for these variables in our analysis as ignoring them may have introduced bias [17].

We estimated remaining lifetime by extrapolating beyond the observed follow-up period using parametric survival models. We explored different distributions (exponential, Weibull, etc.) and assessed goodness of fit by comparing observed cumulative incidence curves/survival probabilities to different model predictions. Although extrapolation is the subject of continuing debate our period of extrapolation was relatively short and the methodology is now well established [18].

## Results

### Epidemiology

We identified 35,208 incident ALD admissions in the period between 1991 and 2011. Overall incident ALD hospitalisation rates are shown in S1 Appendix. Characteristics of cases by severity grouping are shown in Table 3. Definitions of severity groupings are shown in Table 2.

**Table 3:** Characteristics by incident ALD hospitalisation severity grouping.

	Group 1		Group 2		Group 3		All	
Description	with decompensation		without decompensation		with ALD not for ALD			
Number (n) (% of total)	7,330 (20.8%)		12,364 (35.1%)		15,514 (44.1%)		35,208 (100%)	
		% of n		% of n		% of n		% of n
**Sex:**								
Men	5,000	68.2	8,082	65.4	10,926	70.3	24,008	68.2
Women	2,330	31.8	4,282	34.6	4,588	29.6	11,200	31.8
**Age group (years):**								
< 40	675	9.2	1,734	14	1,709	11.0	4,118	11.7
40–49	1,757	24.0	3,174	25.7	3,316	21.4	8,247	23.4
50–59	2,417	33.0	3,632	29.4	4,366	28.1	10,415	29.6
60–69	1,775	24.2	2,745	22.2	3,945	25.4	8,465	24
70+	706	9.6	1,079	8.7	2,178	14.0	3,963	11.3
**SIMD fifths:**								
1 (most deprived)	2,823	38.5	5,154	41.7	6,535	42.2	14,512	41.2
2	1,662	22.7	2,902	23.5	3,558	22.9	8,122	23.1
3	1,200	16.4	1,999	16.2	2,450	15.8	5,649	16
4	853	11.6	1,311	10.6	1,607	10.4	3,771	10.7
5 (least deprived)	712	9.7	899	7.3	1,169	7.5	2,780	7.9
Missing	80	1.1	99	0.8	195	1.3	374	1.1
**Co-morbidity (10 most frequent):**								
Alcohol Related	1,616	22.1	2,734	22.1	5,955	38.4	10,305	29.3
Essential Hypertension	638	8.7	963	7.8	1,868	12.0	3,469	9.9
COPD	460	6.3	880	7.1	1,852	11.9	3,192	9.1
Coronary Heart Disease	360	4.9	657	5.3	1,485	9.6	2,502	7.1
Renal Failure	407	5.6	585	4.7	1,173	7.6	2,165	6.2
Diabetes	313	4.3	488	4	967	6.2	1,768	5
Cancer	344	4.7	277	2.2	911	5.9	1,532	4.4
Cerebrovascular Disease	168	2.3	290	2.4	887	5.7	1,345	3.8
Atrial Fibrillation	203	2.8	313	2.5	815	5.3	1,331	3.8
Chronic Heart Failure	155	2.1	263	2.1	823	5.3	1,241	3.5

Overall, 6,137 patients died during their incident ALD hospitalisation (out of 35,208 incident admissions, 17.4%), comprising 4,113 men (out of 19,895, 17.1%) and 2,024 women (out of 9,176, 18.1%. The results of modelling in-hospital mortality are given in S2 Appendix. The odds of dying in-hospital were higher for the ‘ALD with decompensation’ group (Odds Ratio (OR): 1.75, (95% confidence interval (95% CI) 1.63,1.88, p<0.001) and ‘hospitalisation with ALD (but not for ALD)’ (OR: 1.21, 95% CI 1.20, 1.22, p<0.001) compared to the groups of ‘ALD without decompensation’. There was some evidence of a reduction in in-hospital mortality over the study period with ORs less than 1 since 2003 (OR 2011 vs. 1991: 0.8, 95% CI 0.63, 1.00, p = 0.05).

**Fig 1:**
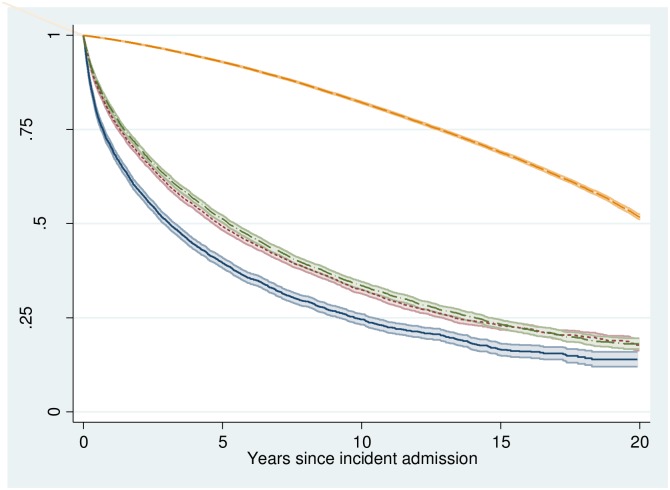
Survival curve by severity group shown with 95% confidence intervals. Solid line—ALD with decompensation, long dash dot—With ALD but not for ALD, short dash—ALD without decompensation and long dash—controls.

The remaining 29,071 (82.6%) patients were discharged alive from their incident ALD hospitalisation. Fig 1 shows survival curves for cases by severity grouping and for controls. For patients with decompensation 5 year survival rates were 40% (95% CI 38%, 41%) and 10 year survival 25% (95% CI 23%, 26%). For patients without decompensation 5 year survival rates were 49% (95% CI 48%, 50%) and 10 year survival 32% (95% CI 31%, 33%). S3 Appendix. shows survival curves by gender by severity group.

The results of modelling all-cause mortality after discharge are shown in S4 Appendix. There was no evidence that the risk of dying after discharge from incident ALD hospitalisation had changed between 1991 and 2011. As for inpatient survival, patients with decompensation at incident admission had a worse outcome if discharged alive with mortality over the full observation period 30% higher in this group (HR: 1.3, 95% CI 1.25, 1.35, p<0.001) compared to patients initially admitted without decompensation. The mean (median) number of readmissions per year (up to death or end of the observation period) for the incident ALD patients who were discharged alive was 3.1 (1.3). The results of modelling readmission rates are shown in S5 Appendix. There was strong evidence that the number of readmissions for patients discharged from an incident ALD hospitalisation in more recent years was greater than for those discharged from incident ALD hospitalisations at the start of the study period (Incidence Rate Ratio (IRR) (2011 vs. 1991) = 1.61, 95% CI 1.43, 1.81 p<0.001).

### Costs

#### Descriptive analysis

The following figures are not adjusted as in the statistical analysis. They are for illustration only.

**Fig 2:**
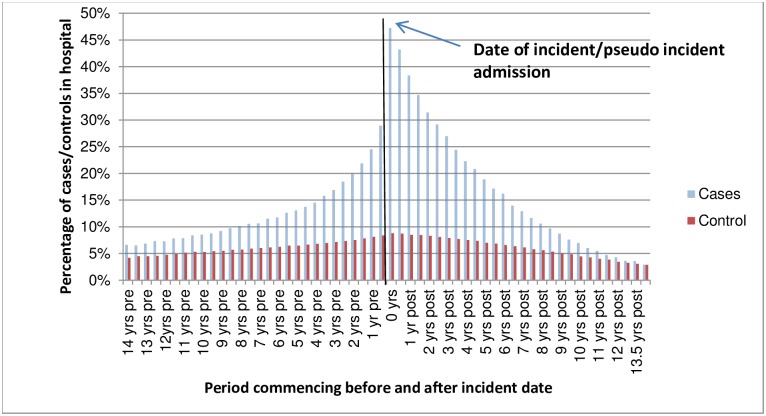
People hospitalised as a percentage of people alive mid-period.

**Fig 3:**
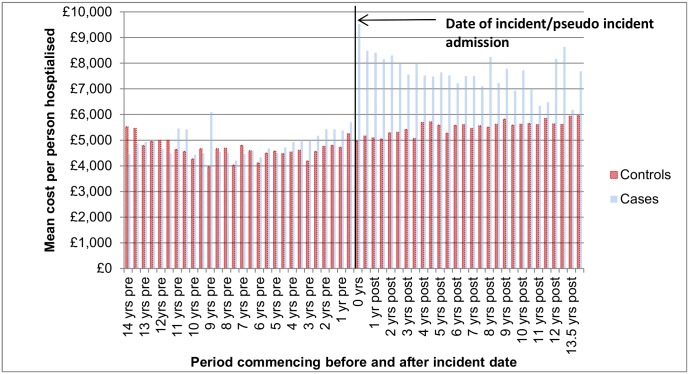
Mean cost (£) per person hospitalised.

**Fig 4:**
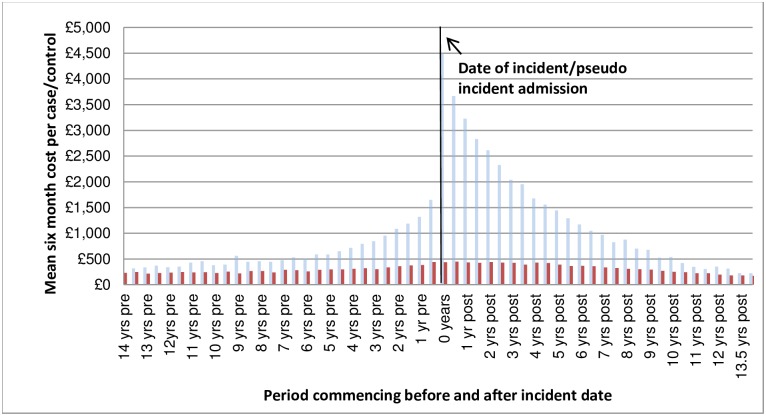
Mean cost (£) per person (whether hospitalised or not).

Fig 2 shows the percentage of cases and controls who had at least one hospitalisation in a given 180 day period before and after the incident ALD hospitalisation. Approximately 29% of the cases were hospitalised in the 180 days before the event and approximately 25% of the cases were hospitalised between 181–360 days before the event. A large proportion of these were alcohol-related (AR) hospitalisations– 21% of the incident ALD cohort had an AR hospitalisation in the year prior to their index admission. As these figures exclude the incident admission itself, the 47% of cases in hospital in the 180 days following the incident admission were readmissions. Fig 3 shows that for those hospitalised, costs for cases were 5% higher in the pre-incident period and 39% higher in the post incident period. Again this figure excludes the incident admission which, if included, would increase the differential between cases and controls. Fig 4 shows the average cost per person in each 180 day period excluding the incident admission cost.

#### Statistical analysis

Table 4 summarises predicted mean and median costs for cases and controls in the pre-incident period, for the incident admission and in the post-incident period. Annual costs are used for the pre and post periods as cases and controls had different periods of exposure during that time. Pre-incident costs were broadly similar for men and women and mean annual costs for cases were 2.4 times those for controls. Mean incident costs were £6,663 (95% CI £6,511, £6,661) with costs for women 13% higher than those for men. In the post-incident period mean annual costs for cases were 10 times those for controls with costs for men 11.9% higher than those for women. Examples of remaining lifetime costs for costs and controls are also summarised in Table 5. Despite shorter life expectancy patients admitted with ALD incur substantial additional costs over their remaining lifetimes when compared to controls. For a man under 40 years old from the least deprived socio-economic category diagnosed with ALD compared to a matched control additional lifetime hospital costs were estimated to be £47,220 (with a difference in life expectancy of 30 years). For a woman over 70 years old from the most deprived socio-economic deprivation category the additional costs were estimated to be £124,716 (with a difference in life expectancy of 7 years). Confidence intervals around all costs estimates are narrow due to the large number of observations in this study.

**Table 4:** Summary of costs for cases and controls (based on predicted costs from modelling output).

	Cases				Controls				Difference	
	Mean	Median	95% CI		Mean	Median	95% CI		Mean	Median
	£	£	Lower(£)	Higher(£)	£	£	Lower(£)	Higher(£)	£	£
Pre-incident period	1,371	1,269	1,370	1,372	567	522	567	567	804	747
(costs per annum)										
Men	1,365	1,258	1,364	1,366	565	519	565	565	800	739
Women	1,385	1,276	1,384	1,386	572	524	572	572	813	752
Incident admission	6,663	6,511	6,661	6,665	N/A	N/A	N/A	N/A	N/A	N/A
(costs per CIS of initial ALD diagnosis)										
Men	6,396	6,259	6,393	6,399	N/A	N/A	N/A	N/A	N/A	N/A
Women	7,242	7,076	7,238	7,246	N/A	N/A	N/A	N/A	N/A	N/A
Post-incident period	14,196	10,271	14,182	14,210	1,422	1,036	1,421	1,423	12,774	9,235
(costs per annum)										
Men	14,689	10,843	14,671	14,707	1,472	1,092	1,406	1,538	13,217	9,751
Women	13,127	9,289	13,103	13,151	935	1,313	869	1,001	12,192	7,976

**Table 5:** Estimate of remaining lifetime costs.

Remaining lifetime costs	Case					Control					Difference
	Annual mean	95% CI		Remaining	Remaining	Annual mean	95% CI		Remaining	Remaining	Remaining
	cost(£)	Lower(£)	Higher(£)	life(years)	life cost(£)	cost(£)	Lower(£)	Higher(£)	life(years)	life cost(£)	life cost(£)
Most deprived—men <40	5,401	5,394	5,408	17.3	93,599	529	529	529	38.3	20,261	73,339
Least deprived—men <40	3,515	3,501	3,529	18.2	63,832	349	348	350	47.6	16,612	47,220
Most deprived—men 50–59	12,474	12,464	12,484	7.4	92,682	1,224	1,224	1,224	21.8	26,683	65,999
Least deprived—men 50–59	7,812	7,797	7,827	8.2	63,902	770	769	771	28.0	21,560	42,342
Most deprived—men 70+	46,939	46,866	47,012	3.5	165,695	4,608	4,605	4,611	9.3	42,854	122,840
Least deprived—men 70+	29,109	29,030	29,188	4.1	119,056	2,863	2,859	2,867	12.2	34,929	84,127
Most deprived—women <40	4,993	4,984	5,002	18.6	92,770	493	493	493	43.2	21,298	71,472
Least deprived—women <40	3,216	3,196	3,236	19.7	63,323	316	315	317	49.7	15,705	47,618
Most deprived—women 50–59	11,781	11,766	11,796	8.2	96,840	1,154	1,153	1,155	25.3	29,196	67,644
Least deprived—women 50–59	7,528	7,508	7,548	9.2	68,881	734	733	735	30.5	22,387	46,494
Most deprived—women 70+	42,817	42,707	42,927	4.0	171,268	4,232	4,227	4,237	11.0	46,552	124,716
Least deprived—women 70+	27,742	27,634	27,850	4.6	128,168	2,741	2,736	2,746	13.4	36,729	91,439

The results of modelling daily pre-incident and post-incident hospital costs per person are shown in Appendices 6 and 7 (SM1). These costs are calculated by adding the costs of all the admissions in the observation period (excluding incident costs) and dividing by the number of days of observation. In the pre-incident period the mean daily cost for cases was 2.4 times that for controls (IRR 2.44, 95% CI 2.26, 2.62, p<0.001) and 10.2 times in the post-incident period (IRR 10.15, 95% CI 9.56, 10.78, p<0.001). There was a strong socio-economic gradient in both periods—costs for patients from the most deprived areas were 51% higher in the pre-incident period (IRR 1.51 95% CI 1.36, 1.67, p<0.001) than those from the least deprived areas and 57% in the post-incident period (IRR 1.57, 95% CI 1.45, 1.71, p<0.001). Costs showed a strong age gradient and daily costs for the 70+ age category were 2.8 times those of the under 40s in the pre-incident period (IRR 2.81, 95% CI 2.51, 3.16, p<0.001) and 8.7 times in the post-incident period (IRR 8.68, 95% CI 7.91, 9.53, p<0.001).

The results of modelling overall incident admission costs (ie cost of the CIS when ALD was first diagnosed) are shown in S8 Appendix. The results show a similarly strong age gradient to pre incident costs but there is no evidence that socio-economic deprivation category affected incident costs. Costs for women were 15% (IRR 1.15, 95% CI 1.10, 1.21 p<0.001) higher than for men. Incident costs were 26% (IRR 1.26, 95% CI 1.18, 1.33, p<0.001) higher for those patients with decompensation compared to those without.

There is strong evidence that both overall incident admission and daily post admission costs have reduced over the study period. Incident costs show a 43% reduction (IRR 0.57, 95% CI 0.48, 0.67, p<0.001) and post incident costs a 28% reduction (IRR 0.72, 95% CI 0.61, 0.85 p<0.001) between 1991 and 2011. Descriptive analysis suggests that these reductions have mainly been driven by a reduction in length of stay which has reduced from a median of 10 to 5 days for incident admissions. The mean length of stay for all admissions has reduced from 9.4 days to 4.6 days for cases and from 6.2 days to 3.8 days for controls (S9 Appendix).

## Discussion

This study aimed to identify hospital admissions and associated costs before and after diagnosis and outcomes for all individuals hospitalised for the first time with ALD in Scotland between 1991–2011 compared to population controls matched by age, sex and socio-economic deprivation.

### Main results

#### Epidemiology

Our results were broadly consistent with previously published statistics and studies of ALD. Incidence rates identified in the study matched previously published data for Scotland [19, 20]. The risk of dying in-hospital during an incident ALD hospitalisation for this cohort was 17.4% overall with higher rates for admissions because of ALD decompensation (23%). There was evidence that hospital mortality rates had reduced over the study period (OR 0.8, 95% CI 0.63, 1.0, p = 0.05), suggesting a 20% reduction. Survival rates were poor and we found no evidence of an improvement in survival rates post-discharge over the study period. Readmission rates post index admission were high and there was evidence that this had increased substantially over the study period.

#### Costs

This study found substantial additional costs associated with every incident ALD admission compared to matched controls. In addition to a mean cost of incident admission of £6,663, cases were 2.1 times more likely to have been in hospital in the pre-incident period costing an average of £804 per annum more than matched controls over the 14 years preceding the incident admission. In the period following the incident admission annual costs associated with cases were over 10 times those of their matched controls. Even with shorter life expectancies remaining lifetime costs were substantially higher for cases than controls with strong age and socio-economic gradients. The study found strong evidence that the cost of both incident and individual post-discharge admissions has reduced over the study period. This reduction seems to have been driven mainly by reduced length of stay with median incident length of stay reducing from 10 to 5 days over the study period and substantial reductions in all lengths of stay for cases and controls.

### Strengths of our approach

This was a population-wide study using data at an individual level which permitted analysis taking age, sex and socio-economic factors into account. We had the benefit of a large sample size and detailed follow-up over a long period was possible through data linkage of high quality records. As Scotland benefits from a universal healthcare system free at the point of delivery, selection bias was minimised. The matched cohort design of the study together with the inclusion of the matched variables in the statistical analyses (age, sex and socio-economic deprivation category) provided assurance that unmeasured confounding was minimised. The categorisation of admissions undertaken in this study allowed analysis of outcomes and costs by disease severity.

### Limitations of our approach

A limitation to our study is that incident cases were identified from hospital records only and that cases of diagnosed ALD which did not lead to hospitalisation were not included in our analysis. We obtained data on ALD deaths without a hospital admission. There were 5,320 over the full study period (which, had they been admitted to hospital could have increased our overall incidence by 13.1%). However, these were not included in our analysis as they did not incur hospital costs. Our analysis did not include emergency hospital attendances where those did not lead to a hospital admission as these are excluded from the data sources used (SMR01 and SMR04). A further limitation of the study is that only hospital costs were included and not costs incurred in primary or social care. The majority of care for ALD occurs in hospital [10] and the inclusion of primary care costs would be likely to increase the additional costs associated with an incident ALD diagnosis. The inclusion of primary health care data may have altered our findings in other areas of the study, such as survival, as it is likely that the patients identified through hospital episodes were the most severe.

A limitation of our study design is that controls were only matched on sex, age and socio-economic status. We did not match on comorbidity and we did not adjust for it in the cost analyses as we wished to capture the full difference in costs between cases and controls although we hypothesised that much of the additional co-morbidity would have resulted from excess alcohol consumption and therefore should not have been adjusted for. We were unable to adjust for tobacco consumption despite the likelihood that smoking was more prevalent among cases. We are therefore unable to attribute the full additional cost of ALD cases to the ALD diagnosis.

Our method for calculating lifetime costs is based on a modelled daily cost which is then multiplied by an estimated remaining lifetime. It is possible that high costs for severe cases who died quickly may have resulted in higher average costs particularly when extrapolated over a long period (as for the younger age categories). However this possible overestimation might be partially offset by expected increasing inpatient care as people age and are nearing the end of life. Future research should examine in more detail the treatment pathways for ALD post diagnosis in order to better understand the main drivers of costs.

### How our findings fit with the existing literature

#### Epidemiology

Our findings on incident rates were generally consistent with and have been superceded by the most recent national data released for Scotland [19,20]. The Monitoring and Evaluating Scotland’s Alcohol Strategy (MESAS) Final Annual report [20] indicated that incidence rates of ALD decreased further to 48 and 20 per 100,000 per year population for men and women respectively in the year to April 2013 but have increased for the years to April 2014 and 2015. The in-hospital mortality risk found in this study (17.4%) is not directly comparable with other studies for various reasons such as not differentiating between index admissions and other ALD admissions [21], including all liver disease [8] and including only patients in acute care [22–24]. A US study reflected our finding of in-hospital deaths reducing over time [10] although their study concerned alcoholic hepatitis. They speculated that the observed reduction from 10.1% in 2002 to 5.8% by 2010 may have been achieved through better management of fluids and infections. A recent Danish study also found decreasing rates of mortality for alcoholic cirrhosis, although this appears to include both in-hospital and post-discharge mortality [25]. The lack of socio-economic gradient in in-hospital mortality is contrary to our findings in other aspects of the study which may suggest that once admitted patients are treated equally with equal outcome up to discharge.

The high rate of in-hospital mortality for incident ALD hospitalisations reflects a number of current issues reported in the literature. Difficulties in the early detection of ALD mean that many patients present late to hospital and their disease is already at an advanced stage [8]. A review of patients who died of ALD in England in 2013 found that many areas lacked specialist care and that less than half of patients had received good care in hospital [26]. Added to these issues are concerns around lack of options for treatment of ALD [27,28] and lack of research highlighted in the European Association for the Study of the Liver (EASL) Clinical Practical Guidelines [5].

Our 5/10 year post-discharge survival rates for patients with ALD decompensation at index admission (40%/25%) were broadly comparable with mortality rates reported in recent studies [29–32]. In contrast to the trend in in-hospital mortality the study found no evidence of a reduction in post-discharge all-cause mortality. Factors such as lack of specialist care and lack of treatment options are also likely to impact on patients post-discharge. For post discharge all-cause mortality those living in more deprived areas had higher mortality rates than their counterparts in less deprived areas.

This study found substantially higher costs associated with incident ALD cases compared to matched controls, driven mainly by the number of days in hospital rather than differences in daily rates. We are not aware of any other studies that look at costs per incident case of ALD. Two US studies estimated costs per hospital admission for alcoholic hepatitis [9,10] reported high and increasing costs driven by increasing daily rates rather than lengths of stay. Many other studies report population level estimates of annual costs of liver disease and/or alcohol-related conditions [eg 7,8] and are difficult to compare to our findings in any meaningful way.

Costs for both cases and controls reduced over the study period driven primarily by reductions in lengths of stay, reflecting national policy in this area ([32], S9 Appendix). The number of readmissions per year increased over the study period with extremely high increases for readmissions for reasons unconnected to alcohol or liver. This reflects ISD data for Scotland which found that the increase in hospital stays 2007–2015 was mainly due to repeat visits from previous patients [33] as well as the findings from two UK based studies [21,34].

#### Importance for policy, practice and future research

Incident ALD hospitalisation rates are high by international standards and although they had reduced at the end of our study period they are now increasing again. Policy makers need to ensure that efforts to reduce population level harms related to alcohol are maintained. ALD disproportionately affects those who live in more deprived areas so effective policies have the potential to reduce health inequalities.

ALD is associated with high in-hospital mortality although this has reduced over time. Further research would be useful to determine why in-hospital mortality is so high, what factors have led to improvements in mortality and the impact of the reducing length of incident stay. Our study used a novel definition of severity in ALD, which has informed our results. These groupings may prove useful in future studies.

Post-discharge survival is poor and has not improved over time. The National Confidential Enquiry into Patient Outcome and Death [26] found a lack of treatment options and suboptimal care offered to suffers of liver disease compared to other chronic conditions in England and the poor prognosis of patients may indicate that the same is true in Scotland. The substantial increase in readmissions merits further investigation as it may be linked to reduced lengths of stay (possibly with insufficient consideration of psychiatric needs on acute medical wards) or poor care in the community.

This study established that patients diagnosed with ALD have had an increased number of hospital admissions for a long period of time before their diagnosis and many of those admissions were for alcohol-related conditions. This would suggest that there is opportunity for early assessment of risk of developing ALD and early clinical intervention which should be investigated further. The high cost of hospital care for each incident case of ALD suggests that effective preventive measures or early treatment options are highly likely to be cost-effective. Alternative treatment pathways post index admission such as assertive outreach or improved palliative care [35] should also improve outcomes and/or quality of life.

The new information on costs provided in this paper can be used in future cost-effectiveness analyses. However, a more detailed exercise examining treatment pathways for ALD post diagnosis and associated costs would be beneficial.

## Supporting Information

S1 Appendix Incident rates by gender Men (blue, higher line) Women (red, lower line). (XLSX)

S2 Appendix Modelling of whether patient died during their incident ALD hospitalisation (logistic regression model). (XLSX)

S3 Appendix Survival curves by severity group by gender. (XLSX)

S4 Appendix Modelling of all-cause mortality after discharge from incident ALD hospitalisation (Cox regression model). (XLSX)

S5 Appendix Modelling of rate of readmissions (negative binomial model). (XLSX)

S6 Appendix Modelling pre-incident daily costs (generalised linear model, log link). (XLSX)

S7 Appendix Modelling post-incident daily costs (generalised linear model, log link). (XLSX)

S8 Appendix Modelling incident costs (generalised linear model, log link). (XLSX)

S9 Appendix Length of stay over study period. (XLSX)
